# Analysis and Phylogeny of Small Heat Shock Proteins from Marine Viruses and Their Cyanobacteria Host

**DOI:** 10.1371/journal.pone.0081207

**Published:** 2013-11-12

**Authors:** Halim Maaroufi, Robert M. Tanguay

**Affiliations:** 1 Plate-forme de bio-informatique and Institut de biologie intégrative et des systèmes (IBIS), Université Laval, Quebec, Canada; 2 Laboratory of Cellular and Developmental Genetics, Department of Molecular Biology, Medical Biochemistry and Pathology, IBIS and PROTEO, Université Laval, Quebec, Canada; University of Arkansas for Medical Sciences, United States of America

## Abstract

Small heat shock proteins (sHSPs) are oligomeric stress proteins characterized by an α-crystallin domain (ACD) surrounded by a N-terminal arm and C-terminal extension. Publications on sHSPs have reported that they exist in prokaryotes and eukaryotes but, to our knowledge, not in viruses. Here we show that sHSPs are present in some cyanophages that infect the marine unicellular cyanobacteria, *Synechococcus* and *Prochlorococcus*. These phage sHSPs contain a conserved ACD flanked by a relatively conserved N-terminal arm and a short C-terminal extension with or without the conserved C-terminal anchoring module (CAM) L-X-I/V, suggested to be implicated in the oligomerization. In addition, cyanophage sHSPs have the signature pattern, P-P-[YF]-N-[ILV]-[IV]-x(9)-[EQ], in the predicted β2 and β3 strands of the ACD. Phylogenetically, cyanophage sHSPs form a monophyletic clade closer to bacterial class A sHSPs than to cyanobacterial sHSPs. Furthermore, three sHSPs from their cellular host, *Synechococcus*, are phylogenetically close to plants sHSPs. Implications of evolutionary relationships between the sHSPs of cyanophages, bacterial class A, cyanobacteria, and plants are discussed.

## Introduction

The small heat shock proteins (sHSPs) are a family of stress proteins, found in archaea, bacteria, fungi, plants and animals [1-4]. sHSPs monomers (12-42 kDa) are characterized by a conserved domain of approximately 90 amino acids called α-crystallin domain (ACD), consisting of eight beta strands which form a ß-sandwich fold (Pfam PF00011: Hsp20/alpha-crystallin). This domain is flanked by an N-terminal arm and C-terminal extension variable in both length and sequence between orthologues and may reflect functional specificity and/or preferential chaperone activity [5,6]. sHSPs generally exist as oligomers that are usually polydisperse and change size and organization on exposure to stress and when interacting with substrate [6]. *In vitro* sHSPs have been shown to prevent the irreversible aggregation of non-native proteins during heat shock. Mutations in sHSPs are associated with a variety of severe diseases, including myopathies, dystrophies, and cataracts [7,8]. Phylogenetic analyses indicated that sHSPs were already present in the last common ancestor of prokaryotes and eukaryotes [9,10]. 

Phages are very important in marine systems. They are the most abundant forms of life in the Earth’s oceans with concentrations exceeding 10 million per milliliter of seawater [11]. They influence marine biogeochemical cycles by controlling host abundance and community composition as well as recycling photosynthetically fixed organic carbon as dissolved organic material via viral lysis [12]. Cyanophages infect the marine unicellular cyanobacteria, *Synechococcus* and its sister group *Prochlorococcus* which dominate the picophytoplankton in the oceans [13,14]. To date, the vast majority of phages that are known to infect cyanobacteria are myoviruses [15,16], which are related to phage T4 [17,18]. It has been reported that the sequenced genomes of *Synechococcus* and *Prochlorococcus* phages contain genes with an hsp20/alpha-crystallin domain (PF00011) [18-20].

## Materials and Methods

### Sequence databases, alignment and phylogeny

We searched the presence of sHSPs in the complete sequenced genomes of viruses from the biological databases (GenBank, protein database, and genomes database) using BLASTp, tBLASTn and HMM profile. We have also searched sHSPs in complete sequenced genomes of their host cyanobacteria, *Synechococcus* and *Prochlorococcus*. We aligned sequences of small heat shock proteins (sHSPs) from several species with ClustalW. Secondary structures indicated in the alignment are assigned according to the determined crystal structure of wheat HSP16.9 [21]. GeneBank accession numbers of sequences of cyanophages and cyanobacteria used in this alignment are listed in the Tables 1 and 2, respectively. Phylogenetic tree was constructed using PhyML [22] and BioNJ [23]. Only the ACD and C-terminal extension were used for the phylogenetic analysis. For PhyML, WAG Substitution model and the statistical confidence of the nodes was calculated by aLRT test.

**Table 1:** Cyanophages’ nomenclature.

**Cyanophages**	**Accession number**	**Nomenclature**
Synechococcus phage S-RSM4	YP_003097310.1	HspSP-RSM4*
Synechococcus phage S-PM2	YP_195165.1	HspSP-PM2
Synechococcus phage S-SM1	YP_004323062.1	HspSP-SM1
Synechococcus phage S-SSM5	YP_004324766.1	HspSP-SSM5
Synechococcus phage Syn19	YP_004323990.1	HspSP-Syn19
Synechococcus phage S-SM2	YP_004322303.1	HspSP-SM2
Synechococcus phage S-CBM2	AFK66310.1	HspSP-CBM2
Synechococcus phage S-MbCM6	YP_007001883.1	HspSP-MbCM6
Synechococcus phage syn9	YP_717838.1	HspSP-Syn9
Synechococcus phage metaG-MbCM1	YP_007001660.1	HspSP-MbCM1
Synechococcus phage S-RIM8 A.HR1	YP_007518247.1	HspSP-RIM8
Synechococcus phage S-ShM2	YP_004322832.1	HspSP-ShM2
Synechococcus phage S-SSM7	YP_004324229.1	HspSP-SSM7
Synechococcus phage S-CRM01	YP_004508578.1	HspSP-CRM01
Synechococcus phage S-CAM8	AET72746.1	HspSP-CAM8
Synechococcus phage S-RIM2 R1_1999	YP_007675621.1	HspSP-RIM2
Synechococcus phage S-SKS1	YP_007674470.1	HspSP-SKS1
Synechococcus phage S-CAM1	YP_007673074.1	HspSP-CAM1
Synechococcus phage S-SSM4	YP_007677312.1	HspSP-SSM4
Prochlorococcus phage Syn1	YP_004324522.1	HspPP- Syn1**
Prochlorococcus phage P-SSM4	YP_214702.1	HspPP-SSM4
Prochlorococcus phage P-RSM4	YP_004323305.1	HspPP-RSM4
Prochlorococcus phage Syn33	YP_004323772.1	HspPP-Syn33
Prochlorococcus phage P-SSM2	YP_214406.1	HspPP-SSM2
Prochlorococcus phage P-SSM7	YP_004325000.1	HspPP-SSM7
Prochlorococcus phage P-HM2	YP_004323516.1	HspPP-HM2
Prochlorococcus phage P-HM1	YP_004322573.1	HspPP-HM1

* Hsp for Small heat shock protein; SP for Synechococcus phage and S-RSM4 for strain

** Hsp for Small heat shock protein; PP for Prochlorococcus phage and Syn1 for strain

**Table 2:** Cyanobacteria’s nomenclature and number of genes.

**Cyanobacteria**	**Gene number**	**Accession number**	**Nomenclature**
Synechococcus sp. WH 5701	3	ZP_01083513.1; ZP_01084874.1; ZP_01086483.1	HspS-WH5701.1*; HspS-WH5701.2; HspS-WH5701.3
Synechococcus sp. PCC 7335	3	ZP_05035247.1; ZP_05037140.1; ZP_05039268.1	HspS-PCC7335.1; HspS-PCC7335.2; HspS-PCC7335.3
Synechococcus sp. CB0101	2	ZP_07972696.1; ZP_07973042.1	HspS-CB0101.1; HspS-CB0101.2
Synechococcus sp. CB0205	2	ZP_07971592.1; ZP_07969614.1	HspS-CB0205.1; HspS-CB0205.2
Synechococcus sp. JA-3-3Ab	2	YP_474873.1; YP_475298.1	HspS-JA-3-3Ab.1; HspS-JA-3-3Ab.2
Synechococcus sp. JA-2-3B'a(2-13)	2	YP_477816.1; YP_476514.1	HspS-JA-2-3B'a.1; HspS-JA-2-3B'a.2
Synechococcus sp. PCC 6312	1	YP_007061156.1	HspS-PCC6312
Synechococcus elongatus PCC 6301	1	YP_172414.1	HspS-PCC6301
Synechococcus sp. PCC 7502	1	YP_007106253.1	HspS-PCC7502
Synechococcus sp. PCC 7002	1	YP_001733915.1	HspS-PCC7002
Synechococcus sp. WH 7805	1	ZP_01125036.1	HspS-WH7805
Synechococcus sp. RCC307	1	YP_001228640.1	HspS-RCC307
Synechococcus sp. WH 7803	1	YP_001226126.1	HspS-WH7803
Synechococcus sp. PCC 7336	1	ALWC01000004.1	HspS-PCC7336
Synechococcus sp. RS9917	1	ZP_01079326.1	HspS-RS9917

* Hsp for Small heat shock protein; S for Synechococcus sp. ; WH5701 for strain and .1 for Hsp number

### Molecular modeling and docking

3D models of *Synechococcus* phage sHSP S-MbCM6 (HspSP-MbCM6) and *Synechococcus* sp. PCC 7335.1 sHSP (HspS-PCC7335.1) were constructed using I-TASSER which combines the methods of threading, *ab initio* modeling and structural refinement [24]. Structures of Hsp16.0 from *Schizosaccharomyces pombe* (PDB: 3w1z), Hsp16.9 from *Triticum aestivum* (PDB: 1gme) and αB-crystallin from human (2ygd) were used as templates for HspSP-MbCM6. 3w1z, 1gme and Hsp16.5 from *Methanocaldococcus jannaschii* (PDB: 4eld) served as template for HspS-PCC7335.1. Search of structure similiraty of obtained 3D models was conducted by PDBeFold [25] against PDB database. The electrostatic potential surface of sHSP 3D models was realized with PyMOL software (http://pymol.org/). Pairwise 3D models alignment was performed using Matras software [26]. Docking of the C-terminal extension of cyanophage (HspSP-MbCM6) and cyanobacteria (HspS-PCC7335.1) into hydrophobic pockets of ß4/ß8 strands region revealed by electrostatic potential surface analysis, was conducted by structure alignment to tetramer of wheat Hsp16.9 (PDB: 1gme).

## Results and Discussion

Publications on sHSPs have reported that they are present in archaea, bacteria, fungi, plants and animals but not in viruses. Here, we searched for sHSPs in the complete sequenced genomes of viruses from the biological databases (GenBank, protein database, and genomes database) using BLASTp, tBLASTn and HMM profile. These searches showed that sHSPs are present only in marine viruses (cyanophages) that infect the unicellular cyanobacteria, *Synechococcus* and *Prochlorococcus* (Table 1). We found that the genomes of many, but not all, of these cyanophages contain a single-copy sHSPs gene. Small cyanophage genomes such as *Synechococcus* phage P60 (47872 bp) and *Synechococcus* phage Syn5 (46214 bp) do not contain any sHSP genes. It is interesting to note that *Prochlorococcus* phage P-SSM2 and P-SSM4 lack core T4-like chaperonin genes (rnlA, 31, and 57A), although, both phages contain sHSPs [19]. sHSPs could play the same function as core T4-like chaperonin genes intervening in scaffolding during maturation of the capsid [27]. 

**Figure 1:**
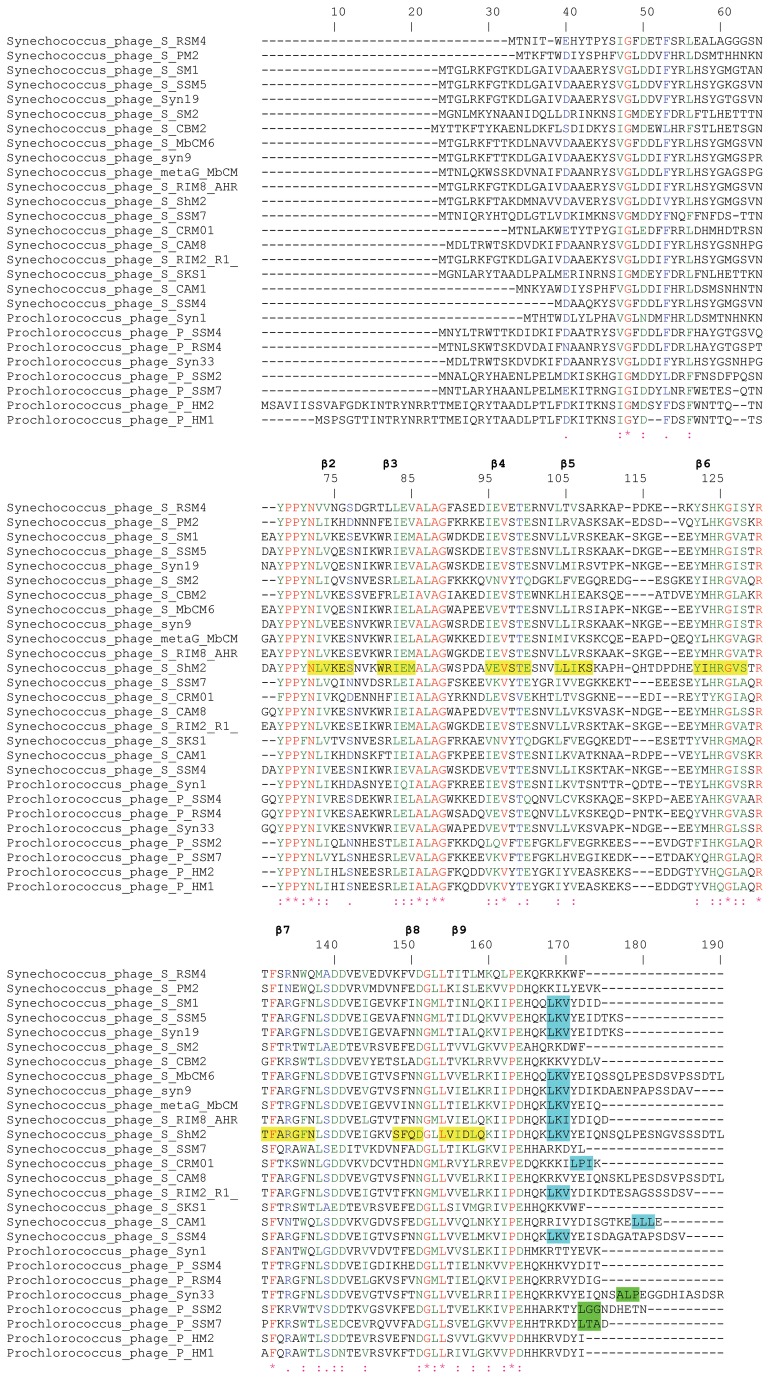
Sequence alignment of cyanophage sHSPs. Amino acids comprising predicted β-strands in *Synechococcus* phage S-ShM2 are in yellow background. The ACD comprises β2-β9. The CAM L-X-I/L/V and non-classical CAM in the C-terminal extension is in cyan and green background, respectively. Alignment was generated using ClustalW. Secondary structures indicated above are assigned according to the crystal structure of wheat HSP16.9 (1gme) [21]. GeneBank accession numbers of sequences used in this alignment are listed in the Table 1.

**Figure 2:**
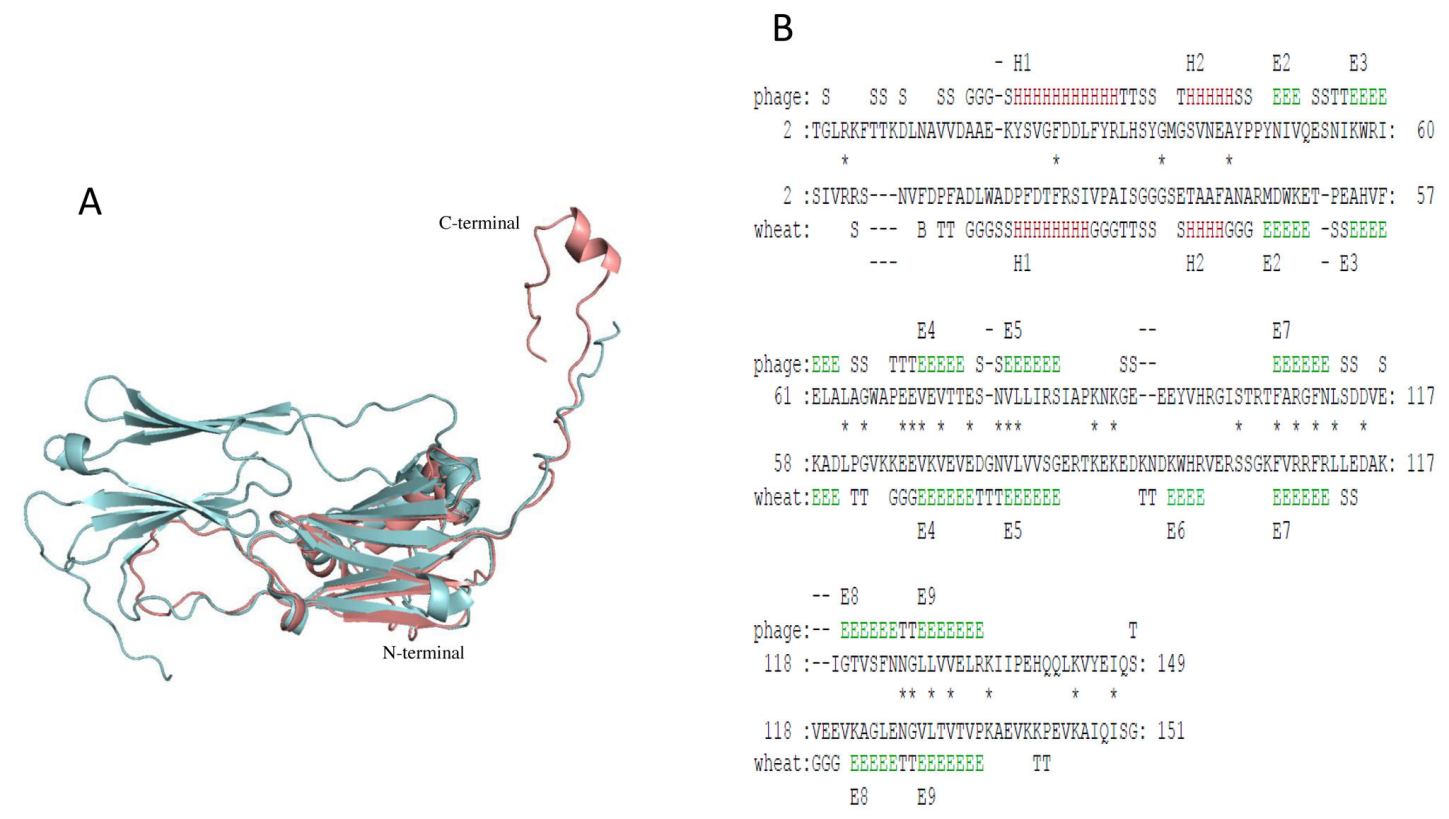
Superposition of 3D structure. **A**. 3D model of cyanophage monomer (pink) was aligned to a dimer (cyan) of wheat sHSP (PDB: 1gme_AB). **B**. Sequence alignment of 3D model of the cyanophage (above) and wheat sHSP structures (bottom) obtained by Matras software [26]. PyMOL software (http://pymol.org/).

Protein sequence analysis of cyanophage sHSPs showed that they contain a conserved ACD (~ 92 amino acids) flanked by a relatively conserved N-terminal arm and a short C-terminal extension. The length of the arm and the extension is variable. Conserved C-terminal anchoring motif (CAM) L-X-I/L/V, implicated in the inter-dimer interactions is present in 12 of 19 *Synechococcus* phages (Figure 1). The *Prochlorococcus* phages do not contain a classical CAM but A-X-P, L-X-G and L-X-A motives are present in the C-terminal extension of *Prochlorococcus* phages Syn33, P-SSM2 and P-SSM7, respectively. It was reported that sHSP Tsp36 also contains a non-classical CAM, I-X-P [28]. The end of N-terminal arm contains a double conserved proline and another conserved proline is present at the beginning of the C-terminal extension (Figure 1). Furthermore, an A-G doublet characteristic of bacterial class A sHSPs is also present in cyanophage sHSPs [29,30] . This doublet is sandwiched by hydrophobic residues, aliphatic residue L and aromatic F/Y/W. Aromatic residues in this position are found only in bacterial classA and animals sHSPs [29]. Cyanophages also have a conserved arginine, important for dimerization and associated with human diseases in the predicted β7 strand (Figure 1). *Synechococcus* phage S-PM2, S-CAM1 and Prochlorococcus phage Syn1 contain a hydrophilic amino acid asparagine in the place of arginine, and *Synechococcus* phage S-CRM01 contains a lysine. The ACD contains a variable region corresponding to the L57 loop (residues 109-121) (Figure 1). Arg in beta7 strand could form salt bridge with Asp or Glu in the L57 loop (residues 109-121) of the neighbor monomer, probably with Asp or Glu in position 117 (Figure 1). Using I-TASSER, we have constructed a 3D model of the sHSP from *Synechococcus* phage S-MbCM6 (HspSP-MbCM6). Figure 2A shows that 3D model is similar to the structure of wheat Hsp16.9 [21]. 3D structure alignment between HspSP-MbCM6 and wheat Hsp16.9 (Figure 2B) showed that the best conserved region is the ACD domain. 3D alignment by PDBeFold of the 3D model against PDB database revealed a high similarity (RMSD of 1.40 Å and 20% of identity) with 1gme.

**Figure 3:**
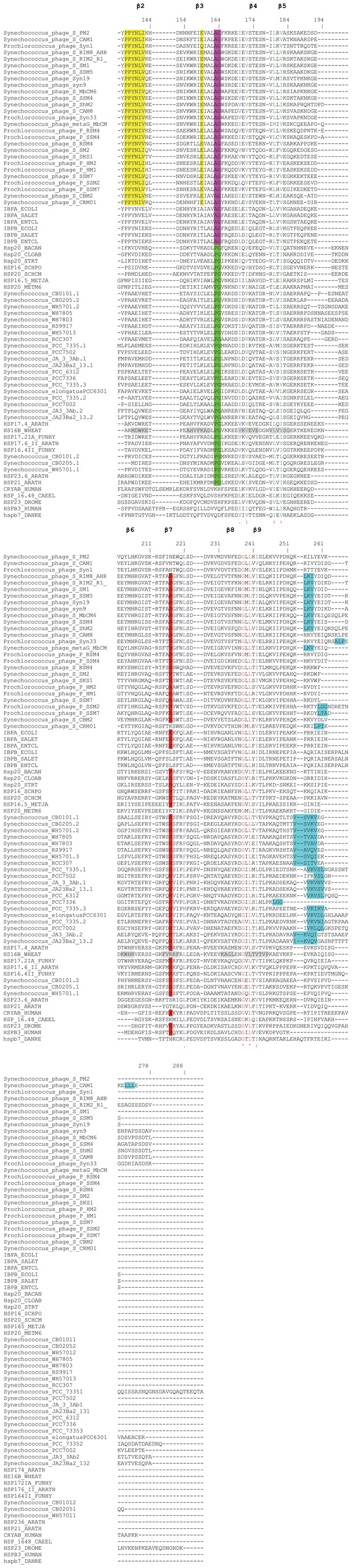
Sequence alignments of cyanophages, prokaryotes and eukaryotes. Amino acids comprising β-strands are in gray background. The ACD comprises β2-β9. The CAM L-X-I/L/V of cyanophages and non-classical CAM I/V-X-I/L/V-X-I/L/V of cyanobacteria in the C-terminal extension is in cyan background. Alignment was generated using ClustalW. Secondary structures indicated above are assigned according to the crystal structure of wheat HSP16.9 [21]. GeneBank accession numbers of sequences of cyanophages and cyanobacteria used in this alignment are listed in the Tables 1 and 2, respectively. IBPA_ECOLI (*Escherichia* *coli* small heat shock protein IbpA, NP_290325), IBPB_ECOLI (*Escherichia* *coli*, NP_290324), IBPA_SALET (*Salmonella* *enterica*, NP_458130), IBPB_SALET (*Salmonella* *enterica*, WP_000605929), IBPA_ENTCL (*Enterobacter* *cloacae*, YP_004949877), IBPB_ENTCL (*Enterobacter* *cloacae*,YP_004949878), Hsp20_BACAN (*Bacillus* *anthracis*, NP_844651), Hsp20_CLOAB (*Clostridium* *acetobutylicum*, NP_350294), Hsp20_STRT (*Streptococcus* *thermophiles*, YP_796431), HSP16_SCHPO (*Schizosaccharomyces* *pombe*, NP_596091), HSP20_SCHCM (*Schizophyllum* *commune*, XP_003031590), HSP16.5_METJA (*Methanocaldococcus* *jannaschii*, NP_247258), HSP20_METM6 (*Methanococcus* *maripaludis*, YP_001548257), HSP17.4_ARATH (*Arabidopsis* *thaliana*, NP_190209), HSP17.6_II_ARATH (*Arabidopsis* *thaliana*, NP_196763), HSP23.6_ARATH (*Arabidopsis* *thaliana*, NP_194250), HSP21_ARATH (*Arabidopsis* *thaliana*, NP_194497), HS16B_WHEAT (*Triticum* *aestivum*, Q41560), HSP17.2IA_FUNHY (*Funaria* *hygrometrica*, AAD09178), HSP16.4II_FUNHY (*Funaria* *hygrometrica*, AAD09184), CRYAB_HUMAN (*Homo* *sapiens*, NP_001876), HSPB3_HUMAN (*Homo* *sapiens*, NP_006299), HSP_16.48_CAEEL (*Caenorhabditis* *elegans*, NP_505355), HSP23_DROME (*Drosophila* *melanogaster*, NP_523999), hspb7_DANRE (*Danio* *rerio*, NP_001006040).

**Figure 4:**
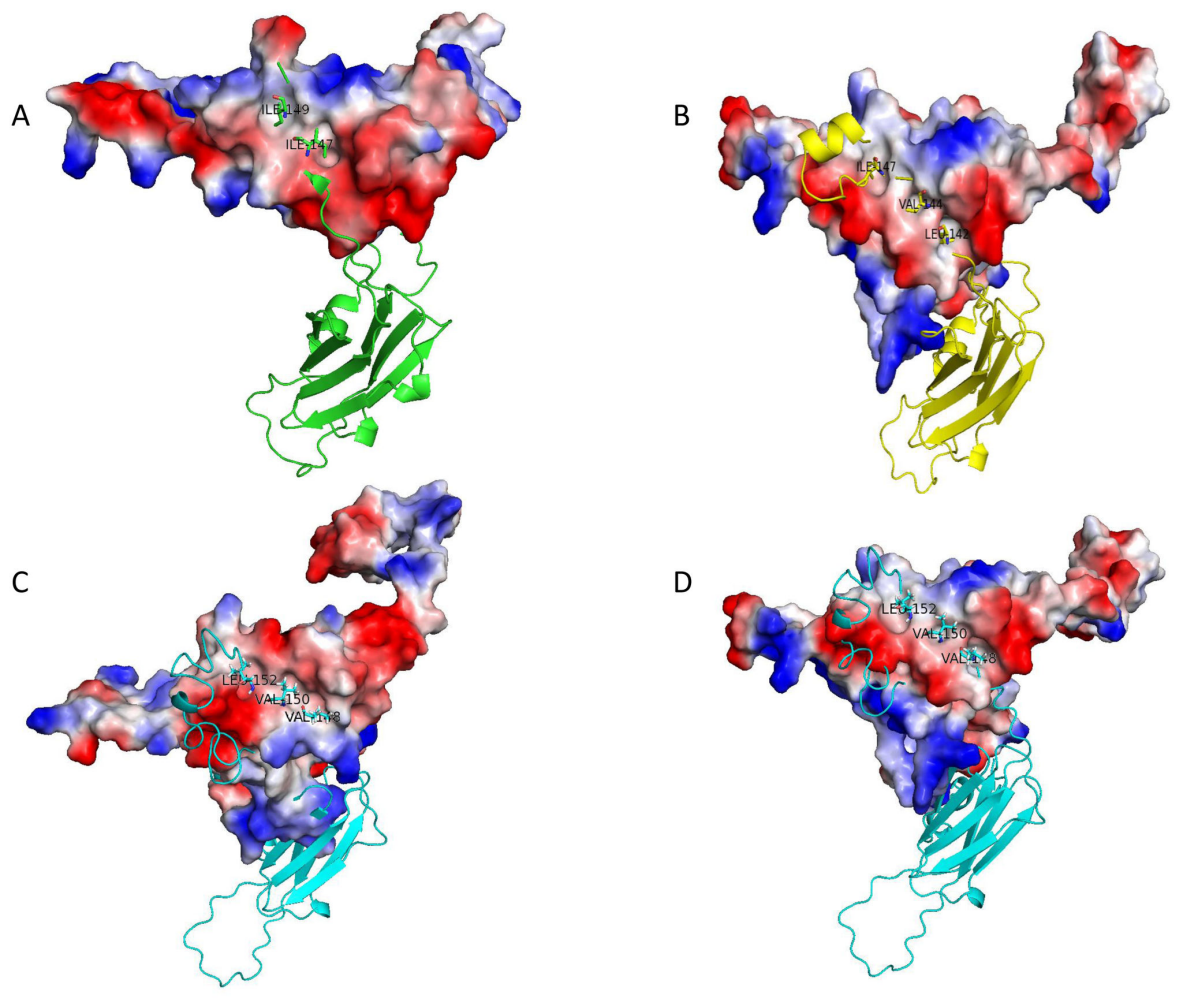
Electrostatic potential surface representation of CAM docking of cyanophages and cyanobacteria into hydrophobic β4 and β8 pockets. **A**. The CAM I-X-I connects dimers in oligomers of wheat Hsp16.9 by interacting with a hydrophobic pockets formed by β4 and β8 (PDB: 1gme_AJ). **B**. Cyanophage dimers interaction . **C**. Cyanobacterial dimers interaction. **D**. Cyanophage-cyanobacteria dimer interaction. The surfaces are coloured by electrostatic potential with negative charge shown in red and, positive charge in blue. For clarity one monomer of each dimer is represented and one monomer is in ribbon form. PyMOL software (http://pymol.org/).

We have also searched for sHSPs in the genomes of their host cyanobacteria, *Synechococcus* and *Prochlorococcus*, in order to know if sHSPs in cyanophages are the result of lateral gene transfer (LGT) from cyanobacteria to phage. LGT from cyanobacteria to cyanophages is well documented for photosynthesis genes [31]. Fifteen sequenced genomes of *Synechococcus* contain 1, 2 or 3 sHSP genes (Table 2) while seven others *Synechococcus* genomes do not. Surprisingly 13 genomes of *Prochlorococcus* do not contain any sHSPs gene. It is possible that genomes of *Prochlorococcus* and some *Synechococcus* have not acquired sHSPs gene by LGT or have lost it. Alignment of cyanobacterial sHSPs (Figure 3) revealed the presence of the P-G doublet characteristic of plants and bacterial class B. It is important to note a novel organization of CAM, three hydrophobic amino acids residues I/V-X-I/L/V-X-I/L/V instead of classical two hydrophobic amino acids residues separated by a non-hydrophobic residue. Moreover, *Synechococcus*_PCC7502 contains the classical CAM (V-X-L) and *Synechococcus*_PCC7336 without CAM (Figure 3). The electrostatic potential surface of 3D models of *Synechococcus* phage S-MbCM6 (HspSP-MbCM6) and the cyanobacteria *Synechococcus* *sp.* PCC 7335.1 sHSP (HspS-PCC7335.1) revealed the presence of three hydrophobic pockets formed by ß4/ß8 strands. Docking of the C-terminal extension into the ß4/ß8 strands grooves revealed that hydrophobic residues L142, V144 and I147 of HspSP-MbCM6 and V148, V150 and L152 of HspS-PCC7335.1 occupied the three pockets (Figure 4B and 4C). These results suggest that sHSPs of cyanophage and cyanobacteria could form hetero-oligomers provided they have compatible N-terminal interactions (Figure 4D). 

**Figure 5:**
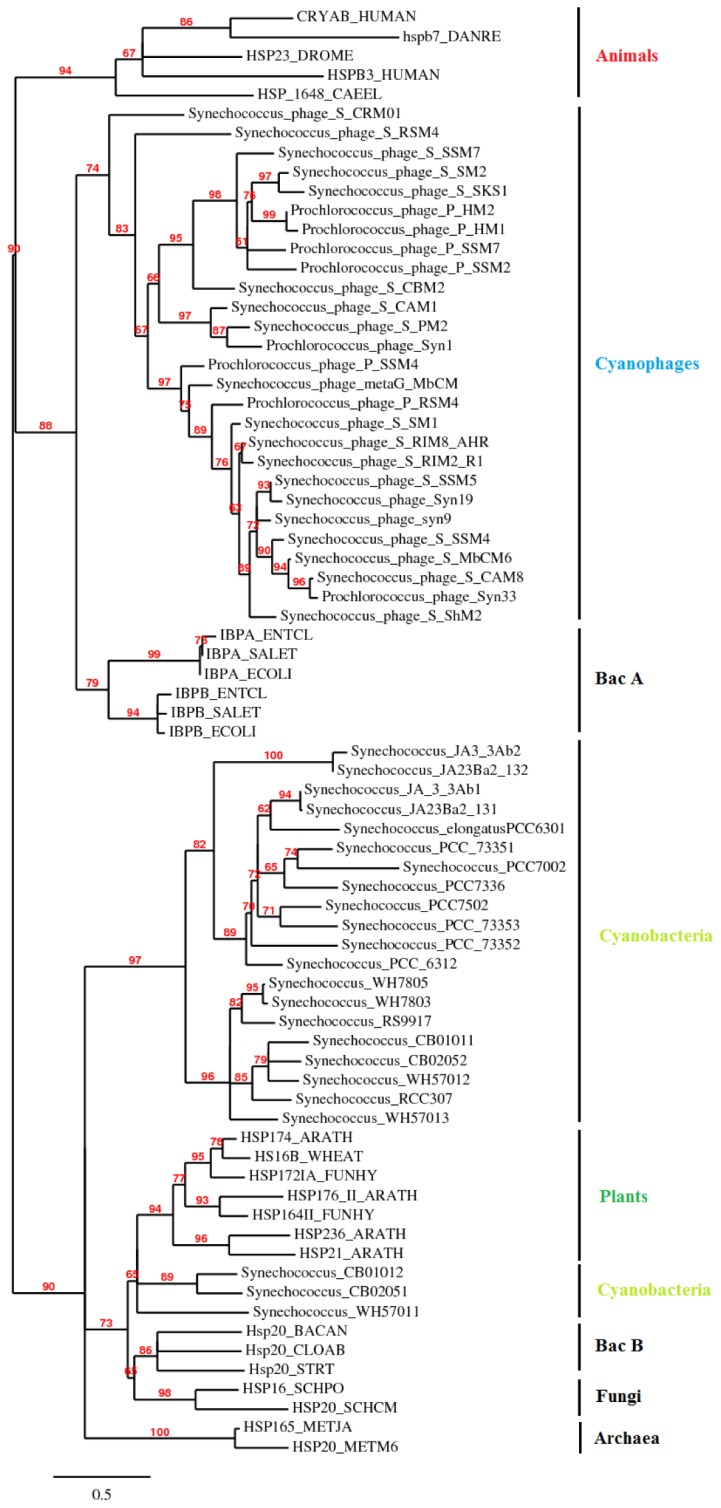
Phylogenetic relations of sHSPs from cyanophages, prokaryotes and Eukaryotes obtained by maximum likelihood. Only the ACD and C-terminal extension were used for the phylogenetic analysis. WAG Substitution model and the statistical confidence of the nodes were calculated by aLRT test. Branches with aLRT values lower than 50% were collapsed.

To establish the phylogenetic relationships between sHSPs of cyanophages and those of prokaryotes and eukaryotes, we aligned sequences of the ACD from bacteria, archaea, cyanobacteria, fungi, plants and animals with ClustalW and constructed a phylogenetic tree using PhyML [22] and BioNJ [23]. The multiple sequences alignment of Figure 3 shows that the pattern P-P-[YF]-N-[ILV]-[IV]-x(9)-[EQ] is a signature of cyanophage sHSPs. This pattern can be used to specifically search for cyanophage sHSPs in metagenomic databases by using PHI-BLAST. Furthermore, the relatively conserved sequences of N-terminal arms of cyanophage sHSPs make it possible to build an HMM profile which can also be employed specifically to extract sHSPs of cyanophages from metagenomic databases. Figure 5 shows that sHSPs form two groups, bacterial class A, cyanophages and animals are one group and bacterial class B, archaea, cyanobacteria, fungi and plants are the second group. The same result is obtained using BioNJ (not shown). In addition, cyanophages sHSPs form a monophyletic clade closer to bacterial class A than to cyanobacteria. This suggests that cyanophages acquired sHSPs gene from a bacterial class A ancestor by LGT. According to the work of Fu et al. [30] based on the relationship between phylogeny and oligomeric polydispersity, we could suppose that cyanophage sHSPs exist in oligomeric polydispersity as in their bacterial class A ancestor sHSP. It is important to note that three sHSPs from their cellular host, *Synechococcus*, form a monophyletic clade that is phylogenetically close to plants (Figure 5). Cyanobacteria are among the most ancient organisms on Earth, and fossils of these photosynthetic bacteria indicate a striking resemblance between current species and ones extant over 2 billion years ago [32]. Thus, the ACD of sHSP gene family must be at least 2 billion years old. We could suppose that plants acquired sHSPs gene from cyanobacterial endosymbionts that gave rise to the chloroplast.

## Conclusions

This study revealed the presence of sHSPs in viruses and highlighted their structural characteristics and phylogenetic relationships with those of prokaryotes and eukaryotes. We expect that the study of sHSPs in a simple system such as viruses and cyanobacteria will help answer many questions not yet resolved such as the mechanism of their interaction with the substrate. Moreover, they could help to know the origin and evolution of this ancient, at least 2 billion years old, gene family.
